# Fatal Progressive Meningoencephalitis Diagnosed in Two Members of a Family With X-Linked Agammaglobulinemia

**DOI:** 10.3389/fped.2020.00579

**Published:** 2020-09-18

**Authors:** Yasushi Kasahara, Masaru Imamura, Chansu Shin, Hiroshi Shimizu, Jirou Utsumi, Ryosuke Hosokai, Haruko Iwabuchi, Takayuki Takachi, Akiyoshi Kakita, Hirokazu Kanegane, Akihiko Saitoh, Chihaya Imai

**Affiliations:** ^1^Department of Pediatrics, Niigata University Graduate School of Medical and Dental Sciences, Niigata, Japan; ^2^Department of Pathology, Brain Research Institute, Niigata University, Niigata, Japan; ^3^Department of Pediatrics, Niigata Cancer Center Hospital, Niigata, Japan; ^4^Department of Pediatrics, Graduate School of Medicine, University of Toyama, Toyama, Japan; ^5^Department of Child Health and Development, Graduate School of Medical and Dental Sciences, Tokyo Medical and Dental University (TMDU), Tokyo, Japan

**Keywords:** X-linked agammaglobulinemia, Bruton tyrosine kinase, progressive meningoencephalitis, neurodegeneration, autoimmunity

## Abstract

Chronic enteroviral meningoencephalitis is a well-known complication in patients with X-linked agammaglobulinemia (XLA). However, progressive neurodegenerative disorders or chronic neuroinflammatory diseases with no causative microorganisms have been recognized as rare central nervous system (CNS) complications in XLA. We herein report a family in which two of three members with XLA had developed progressive meningoencephalitis with an unknown etiology. A 15-month-old male infant presented with left-sided ptosis. Initially, the family denied any family history of inherited diseases, but later disclosed a family history of agammaglobulinemia previously diagnosed in two family members. In the early 1980s, one of the elder brothers of the index patient's mother who had been treated with intramuscular immunoglobulin [or later intravenous immunoglobulin (IVIG)] for agammaglobulinemia deceased at 10 years of age after showing progressive neurological deterioration during the last several years of his life. The index patient was diagnosed with XLA caused by Bruton tyrosine kinase deficiency (654delG; Val219Leufs^*^9), and chronic meningoencephalitis with an unknown infectious etiology. Magnetic resonance imaging of the brain demonstrated inflammatory changes in the basal ganglia, hypothalamus, midbrain, and pons, with multiple nodular lesions with ring enhancement, which showed impressive amelioration after the initiation of IVIG replacement therapy. Pleocytosis, which was characterized by an increase in CD4-positive and CD8-positive T cells expressing an activation marker and an elevation in inflammatory cytokines in the cerebrospinal fluid, was identified. No microorganism was identified as a cause of CNS complications. He thereafter developed brain infarction at 19 months of age and fatal status epilepticus at 5 years of age, despite regular IVIG with high trough levels and regular intraventricular immunoglobulin administration. The etiology of this rare CNS complication in XLA is currently unknown. Previous studies have suggested a possible association of IVIG, which was clearly denied in our index case because of the demonstration of his neurological disorder at presentation. In the future, extensive and unbiased molecular methods to detect causative microorganisms, as well as to investigate the possible role of autoimmunity are needed to clarify the etiology of CNS complications.

## Introduction

X-linked agammaglobulinemia (XLA; OMIM # 300755) is caused by mutations in the *BTK* gene and is characterized by low serum immunoglobulin and defects in B cell maturity, predisposing individuals to severe and fatal central nervous system (CNS) infections (1, 2). While chronic enteroviral meningoencephalitis is a well-known complication in patients with XLA (3–6), progressive neurodegenerative disorders and chronic neuroinflammatory diseases with no identifiable causative infective agents have been recognized as rare CNS complications in XLA (4, 7). However, a limited number of cases have been reported so far, a minimal number of which have information on the clinical course during the early phase of the CNS disease. We have recently encountered a family with XLA, in which two of three family members with XLA were affected with chronic and progressive meningoencephalitis. In the index patient, we were able to closely monitor CNS disease progression from the onset to death.

## Case Report

**Figure 1:**
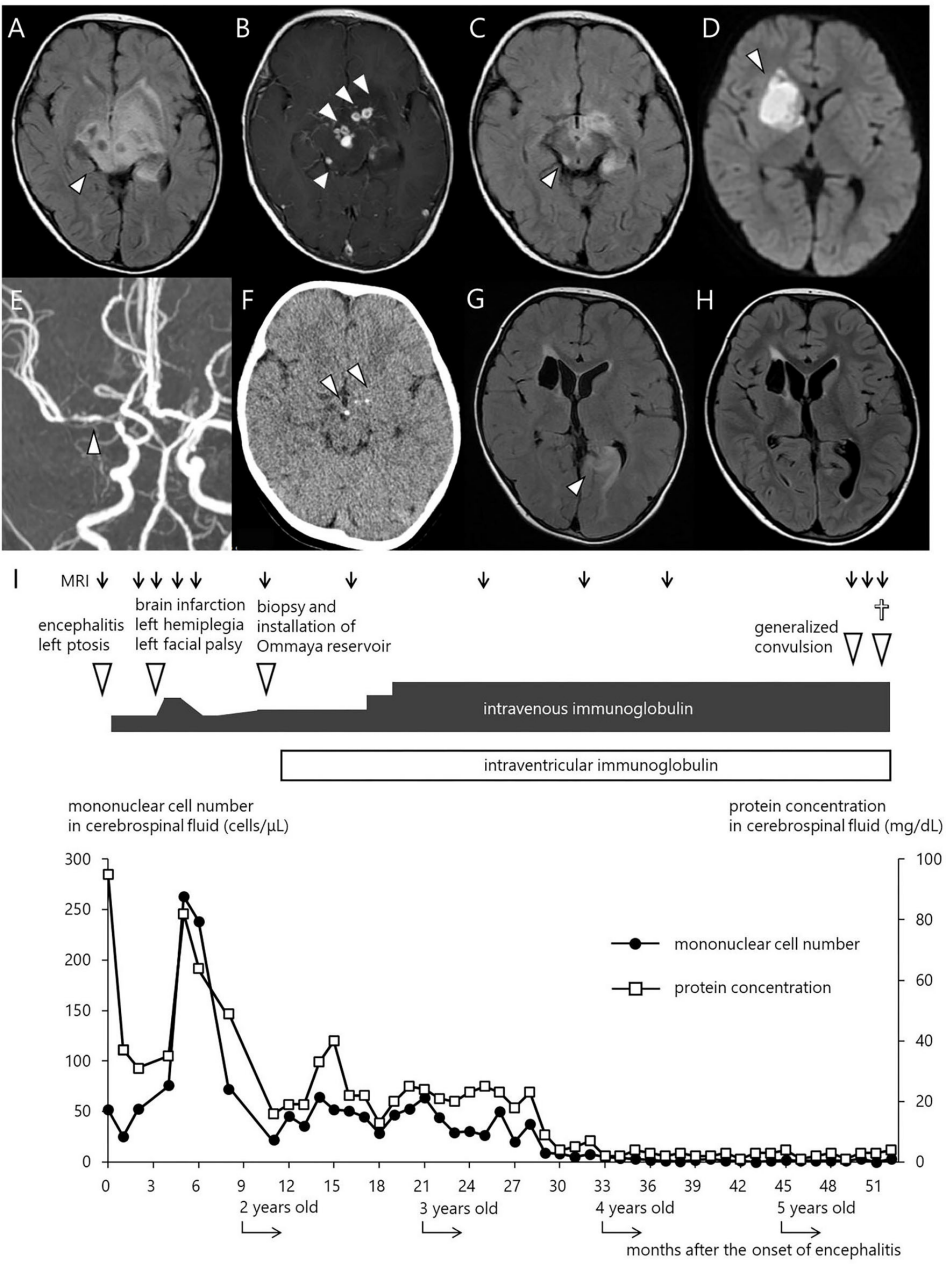
Imaging studies and clinical courses. **(A)** FLAIR MRI scan at initial presentation. **(B)** Contrast enhanced T1 weighted MRI. **(C)** FLAIR MRI 2 weeks after the start of treatment with regular IVIG administration. **(D)** Diffusion weighted MRI at the onset of left facial paralysis and left hemiplegia. **(E)** MR angiography showing a severe stenosis in the right MCA. **(F)** CT scan at 11 months after the start of IVIG. **(G)** FLAIR MRI immediately after the onset of the first generalized convulsion, revealing high signal intensity and swelling in the left occipital legion. **(H)** FLAIR MRI 6 weeks after convulsion. **(I)** Clinical course of the patient. The closed circles and open squares depict the number of mononuclear cells and the level of protein concentration in the CSF, respectively.

The index case was a 15-month-old boy, who presented with progressive left-sided ptosis. His past history was unremarkable with adequate motor and intellectual development (head control at the age of 3 months and walking alone at the age of 13 months). There was no history of intrauterine growth retardation. He had no travel history of going abroad. Initially, the parents denied any family history of inherited disease. He received the BCG vaccine at 4 months, diphtheria-pertussis-tetanus vaccine at 4, 5, and 6 months, and oral polio virus vaccine at 12 and 15 months. Abnormal findings in magnetic resonance imaging (MRI) scans led a local physician to suspect a brain tumor, and the patient was referred to the neurosurgery department in our hospital. The physical examination was not remarkable except for left-sided ptosis, with neither megalocephaly nor craniofacial deformity. The neurological examination was also unremarkable, with neither anisocoria nor facial palsy. Complete blood cell count, blood chemistry including liver function tests and electrolytes, and C-reactive protein (CRP) were within normal ranges. CSF culture was negative. Moreover, serum IgG, IgA, and IgM were undetectable (<10 mg/dL), and no circulating B lymphocytes were detected by flow cytometry. T cells (90.7%) and NK cells (5.6%) were within their normal ranges in numbers. Blast transformation of lymphocytes stimulated with phytohemagglutinin was also within the normal range (stimulation index, PHA 285,629/control 158 = 1807.8; normal range: 101.6–2643.8). The FLAIR images of the MRI brain scan demonstrated a large area with increased signal intensity involving the basal ganglia, hypothalamus, midbrain, and pons, consistent with inflammatory changes (Figure 1A). Contrast-enhanced T1-weighted MRI showed multiple nodular lesions with ring enhancement located in the area (Figure 1B). Spinal MRI and thoracoabdominal computed tomography (CT) showed no abnormal findings. The cerebrospinal fluid (CSF) contained 67 nucleated cells per microliter, 78% of which were mononuclear cells, and an increased total CSF protein of 95 mg/dL.

At this time, the maternal grandmother of the index patient stated that two of her older sons (the patient's uncles) had been diagnosed with hypogammaglobulinemia and died during childhood or adolescence (Supplementary Figure 1). One of them was diagnosed with hypogammaglobulinemia at the age of 2, and was started on intramuscular immunoglobulin replacement at the age of 5. Hearing disturbance and progressive intellectual disability was observed during elementary school. At the age of 9, he developed generalized seizures, and thereafter became unconscious with decorticate rigidity. The CT scan showed marked cerebral atrophy, and he died of progressive neurodegenerative disorder at the age of 10. The autopsy revealed diffuse neuronal loss and gliosis in the cerebral cortex, along with marked perivascular infiltration of lymphocytes and macrophages in the thickened leptomeninges and brain parenchyma. The findings suggested a diagnosis of chronic meningoencephalitis. His younger brother was not reported to have any neurological disorders, but he died of gastric cancer (8) at the age of 20 [previously reported in Japanese (9)]. In the mother and the second brother of the index patient, hereditary exostosis was diagnosed, which required surgical intervention. In the index patient, bone abnormality, including exostosis, was not identified by physical examination. Of note, the two brothers of the index patient had normal levels of immunoglobulins.

The diagnosis of the index patient was confirmed by flow cytometry and a genetic *BTK* test. We found negligible expression of the BTK protein in monocytes by flow cytometry (10), and a single base deletion mutation in exon 8 of the *BTK* gene causing a frame shift and premature termination of the BTK protein (654delG, Val219Leufs^*^9). Overall, we diagnosed him as having XLA, complicated with meningoencephalitis. We conducted an extensive, but inconclusive, search for causative microorganisms, including enterovirus PCR (Supplementary Table 1). Rhinovirus and the poliovirus type 2 vaccine-like strain were isolated from the throat swab and stool, respectively, but not from the CSF, using the viral culture technique. His ptosis began to improve following administration of intravenous immunoglobulin (IVIG) replacement, and disappeared after the 3rd dose of IVIG. Of note, we did not administer corticosteroids or other anti-inflammatory agents. The MRI images rapidly ameliorated (Figure 1C), while repeated lumbar puncture continued to show pleocytosis (Figure 1I). Rhinovirus in the throat and poliovirus vaccine-like strain from the stool became undetectable. As enterovirus is a well-known microorganism associated with chronic progressive meningoencephalitis in XLA patients (3–6), we repeated virus isolation by cell culture as well as PCR several times using the CSF collected by lumber puncture, but failed to show the presence of enterovirus.

At 19 months of age, after an uneventful 4 months with a total of 7 doses of IVIG, he presented with mild fever, left facial paralysis, and left hemiplegia (Figure 1I). An MRI scan revealed a large infarct in the territory of medial lenticulostriate branches of the right middle cerebral artery (MCA) (Figure 1D). MR angiography showed a severe stenosis in the right MCA and in the right anterior cerebral artery (Figure 1E). Levels of antithrombin, protein C, protein S, fibrinogen, and homocysteine were within the normal range, disclosing no thrombotic diathesis. Again, we performed an extensive search for possible pathogens, but failed to identify any potential causative agents (Supplementary Table 1). The cytokine assay of the CSF revealed increased levels of IFN-γ interleukin (IL)-6, and IL-8, in comparison with reference values reported elsewhere (Table 1) (11). He was treated with continuous low molecular weight heparin for 18 days. Doses of IVIG were increased and the interval was shortened to keep trough serum IgG levels higher than 1,500 mg/dL; facial paralysis and hemiplegia gradually recovered after several doses.

**Table 1:** Cytokine and Chemokine levels in the CSF measured at key points in clinical course of this patient.

**Cytokine**	**Reference value (pg/ml)**		**At the onset of hemiplegia (1y9 m)**	**At the installation of Ommaya reservoir (2y3m)**	**At the onset of generalized convulsion (5y5m)**
IFN-γ	6.8	(0–36.84)	60	19.7	5.3
IL-1b	1.6	(0.24–3.9)	<10^*^1	0.3	0.1
IL-6	6.2	(1.35–15)	313	165.6	3.1
IL-8	32.3	(5.4–59)	465	79.0	3.6
TNF-α	2.1	(0–7.5)	5.2	1.4	0.4
IL-1Ra	10.8	(0–17)	n.p.	8.9	1.9
IL-2	0.9	(0.7–9.96)	n.p.	1.9	1.3
IL-4	1	(0–3)	n.p.	0.2	not detected
IL-5	0.5	0.46	n.p.	not detected	not detected
IL-7			n.p.	2.7	2.4
IL-9			n.p.	4.2	2.2
IL-10	5.3	(0–29)	n.p.	1.4	0.2
IL-12	0.4	0.4	n.p.	2.7	2.2
IL-13	4.8	(0–15.13)	n.p.	4.1	2.2
IL-15			n.p.	3.4	1.9
IL-17	1.6	(0–3.72)	n.p.	4.4	4.3
Eotaxin	6.3	(0–13.12)	n.p.	6.0	3.2
FGF basic			n.p.	22.3	12.6
G-CSF	17.3	(0–50)	n.p.	27.1	1.8
GM-CSF			n.p.	49.5	not detected
IP-10	605.1	(45.5–1299)	n.p.	21399.3	355.9
MCP-1	381.1	(136–819.1)	n.p.	136.7	84.8
MIP-1α	4.4	(0.48–6.4)	n.p.	1.2	0.4
PDGF-BB			n.p.	4.7	1.7
MIP-1β	12.1	(4–27.38)	n.p.	28.6	10.2
RANTES	2.7	(2.1–3.16)	n.p.	72.6	not detected
VEGF			n.p.	28.9	8.6

*IFN-γ, interferon-gamma; IL, interleukin; TNF-α, tumor necrosis factor-alpha; IL-1Ra, interleukin-1 receptor antagonist; FGF, fibroblast growth factors; G-CSF, granulocyte-colony stimulating factor; IP-10, Interferon gamma-induced protein 10; MCP-1, monocyte chemotactic protein-1; PDGF, platelet derived growth factor; RANTES, regulated on activation normal T cell expressed and secreted; VEGF, vascular endothelial growth factor; n.p., not performed. Reference values of various cytokines and chemokines in the CSF were reviewed by Kothur et al. (8), and are presented as mean and range in parenthesis*.

* *1, The cytokine levels at the onset of hemiplegia were determined by enzyme immunoassay (EIA) for IFN-γ, enzyme-linked immunosorbent assay (ELISA) for IL-1b, IL-8, and TNF-α, and chemiluminescent enzyme immunoassay (CLEIA) for IL-6. The detection range of the ELISA assay used for IL-1b was 10 pg/ml or higher, which is above a reference value. Therefore, it is unknown whether IL-1b levels at the onset of hemiplegia were within the normal range. The cytokine levels at later points were determined using a cytometric bead array (Bio-Plex Pro Human Cytokine 27-plex assay, Bio-Rad, Hercules, CA, USA)*.

**Figure 2:**
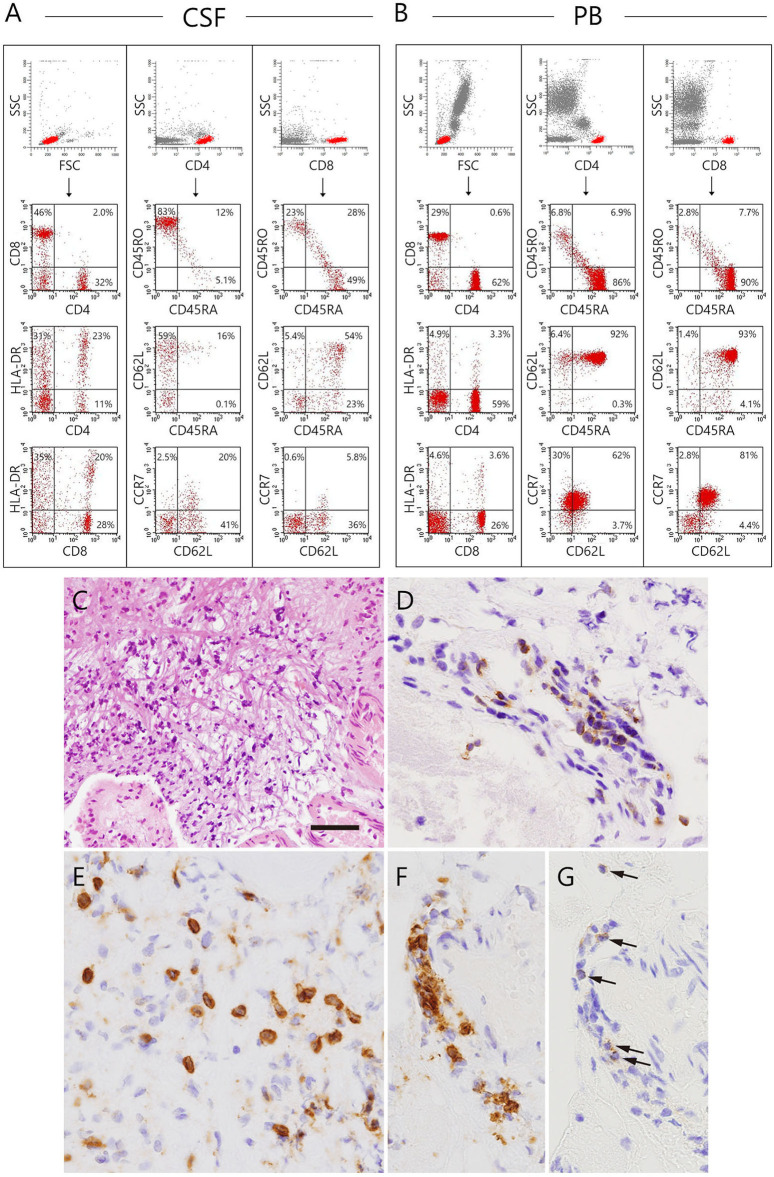
Immunophenotyping of lymphocytes in the CSF and peripheral blood and biopsy specimen of the patient collected at the installation of the Ommaya reservoir. **(A,B)** Dot plot analysis of the CSF and peripheral lymphocytes are shown. The dot-plot in the top of each row was used to gate a population of interest. **(C–G)** The biopsied leptomeninges show fibrous thickening and infiltration of mononuclear cells. **(C)** Hematoxylin eosin staining. **(D–G)** The cells infiltrating in the leptomeninges **(D,E)** and perivascular areas (**F,G** arrows) are positive for several T-cell markers, including CD3 **(D)**, CD8 **(E,F)**, and CD4 **(G)**. Bar: **(C)** 100 μm, **(D–G)** 25 μm.

At 26 months of age (11 months after the start of IVIG), an MRI revealed that the nodules previously detected were reduced in number and size. CT revealed calcification of the nodules (Figure 1F). Although speech delay was present, the patient did not show hearing impairment, dystonia, or gait disturbance. As pleocytosis continued to be present, we then examined mononuclear cells in the CSF collected by lumbar puncture (Figures 2A,B). Activated CD4^+^ T cells (HLA-DR^+^CD4^+^, 22.6%) and activated CD8^+^ T cells (HLA-DR^+^CD8^+^, 19.5%) in the CSF were remarkably increased compared with that in the PB (CD4^+^, 3.3%; CD8^+^, 3.6%). The naïve T cell subset comprised a major fraction of the CD4^+^ T cell population in the PB (CD45RA^+^CD45RO^−^, 86.3%), while the memory T cell subset accounted for a major portion of the CD4^+^ T cells (CD45RA^−^CD45RO^+^, 82.6%) in the CSF. The percentage of memory T cell subsets in the CD8^+^ lymphocytes in the CSF (22.7%) was also much higher than in the PB (2.8%). These results led us to hypothesize that there could be an unidentified pathogen that persistently infected the CNS, or existence of autoreactive T cells that attack brain tissues, and that a higher immunoglobulin concentration in the CNS might exhibit better therapeutic effects. With the approval of the local ethics committee, we initiated monthly intraventricular immunoglobulin administration (100 mg/dose initially, increased up to 500 mg/dose) together with high dose IVIG (1,200 mg/kg to maintain trough IgG levels of 1,500 mg/dL). During the operation for implantation of an Ommaya reservoir, we biopsied the leptomeninges covering the parietal lobe. The pathological findings of the specimen included fibrous thickening with infiltration of CD4^+^ and CD8^+^ T cells (Figures 2C–G). Again, we performed an extensive search for the presence of possible pathogens in the leptomeninges as well as the CSF, by the use of whole-genome amplification and direct sequencing techniques (rapid determination of viral RNA sequences [RDV] method) (12) in addition to conventional PCR assays, but failed to identify a causative pathogen.

At 5 years of age (38 months after the start of intraventricular immunoglobulin), while he attended preschool, he developed an afebrile, brief, generalized seizure for the first time. Low copies of varicella zoster virus (VZV) DNA were identified in his serum by real-time PCR assays (Supplementary Table 1), but not in the CSF. He had neither a history of chickenpox nor of VZV vaccination. Moreover, he had no skin lesions and no signs suggestive of pneumonia. The FLAIR MRI images of the brain revealed a new lesion of high signal intensity and swelling in the left occipital legion (Figures 1G,H), suggesting the ischemic change in the brain might have been caused by vasculitis due to VZV infection. After 10 days of intravenous acyclovir administration, serum VZV became undetectable by PCR. However, after 2 months, he developed a second generalized seizure and deep coma. He was treated in our emergency room, but did not respond to any treatment and died. Request of autopsy was denied by the parents.

## Discussion

In this study, we have reported a family in which two of three members with XLA developed fatal progressive meningoencephalitis with an unknown etiology. In the index patient, extensive searches, which were performed at the initial presentation and during the progression of his CNS lesions, failed to show the presence of any causative microorganism in the serum, CSF, and leptomeninges. None of the current molecular techniques, including PCR, had been available in the early 1980s when the maternal uncle of the index patient died of the disease at 10 years of age. In the index case, the neuroinflammatory disorder developed before the initiation of regular IVIG, excluding the etiological role of immunoglobulin reagents. Infiltration of CD4^+^ and CD8^+^ T cells in the leptomeninges with concomitant increase of activated CD4^+^ and CD8^+^ T cells and increased levels of inflammatory cytokines, including IFN-γ, IL-6, and IL-8, in the CSF were observed. To the best of our knowledge, this is the first report describing XLA patients with chronic meningoencephalitis in the same family. This study is also the first to investigate the cytokine profiles and *in situ* T cell activation, differentiation, and expansion in the CSF of a patient with XLA and chronic meningoencephalitis.

Progressive neurodegeneration with no identifiable infectious disease in patients with XLA and other primary immunodeficiency has been previously reported (7, 13–19). We assessed 14 patients from eight studies performed over the past 20 years (Table 2), by searching Pubmed using a combination of words, including X-linked agammaglobulinemia and neurodegeneration, or neurodegenerative, or neurological, or encephalitis, or meningitis, or meningoencephalitis. An abstract was identified by Google with the words X-linked agammaglobulinemia and neurodegeneration. The median age of diagnosis of neurological disease was 10.0 years (range: 1.3–20). All patients developed their neurological symptoms after initiation of regular IVIG, except for the index patient in this study. The final prognosis was generally poor, with nine deaths out of 13 patients with a known outcome. Similar neurological complications without identifiable microorganisms have been reported in patients with CD40 ligand deficiency (X-linked hyper IgM syndrome), which causes severe impairment of antibody production (20). Five patients with neurodegeneration were identified from a cohort of 31 patients with a molecular diagnosis of CD40 ligand deficiency, all of whom were on regular IVIG. Three of the patients died of neurodegeneration (20). The fact that similar neurological diseases occurred in primary immunodeficiency syndromes showing impaired immunoglobulin production (7, 20) indicates that infection should be the primary cause. Boys with XLA are uniquely susceptible to infection with encapsulated bacteria and enteroviruses (1, 2). Chronic enteroviral meningoencephalitis in agammaglobulinemia (CEMA) is a well-known CNS complication in XLA (3, 4). In the index patient of this study, however, enteroviruses were not identified despite repeated virus isolation assays (cell culture) and PCR assays. Intracranial calcification was found 4 months after diagnosis and the initiation of the regular IVIG treatment. Intracranial calcification can be caused by CNS infections of various pathogens, such as tuberculosis, varicella, and herpes simplex virus (21). Similarly, cerebral calcification was observed in the context of intrauterine infection with cytomegalovirus, rubella, and toxoplasma (21). We attempted to detect these pathogens at multiple stages throughout the disease progression in the index patient, but were unable to successfully identify the causative agent. In two adolescents with XLA and progressive encephalitis, astrovirus, a single-stranded enteric RNA virus causing mild gastroenteritis in humans, had been identified in the brain tissue by the use of novel molecular technologies, including unbiased high-throughput pyrosequencing and next generation sequencing (22, 23). Cache valley virus, a mosquito-borne orthobunyavirus, detected throughout North America, Central America, and parts of South America (24), was identified by the use of metagenomic next generation sequencing in the CSF and brain biopsy tissue in an Australian adult patient with XLA who developed chronic meningoencephalitis after travel to the United states (25). To investigate the etiological microorganism in our index patient, we utilized the RDV method (12, 26), an unbiased molecular method that detects RNA viruses, but failed to identify any causative viruses.

**Table 2:** Progressive neurodegeneration without infection in patients with XLA.

	**Year of report**	**Patient #**	**Previous IVIG**	**Age of diagnosis (years)**	**Age of neurological onset (years)**	**CSF pleocytosis**	**Death**	**References**
1	2002	4	4/4	2, 1.5, 2, 1	5, 4, 11, 14	Not reported^*^	3/4	(7)
2	2004	1	1/1	0.5	5	1/1	1/1	(13)
3	2007	1	1/1	1	14	0/1	1/1	(14)
4	2011	2	2/2	3, 3.5	6, 4	Not reported	2/2	(15)
5	2012	1	1/1	5	14	0/1	0/1	(16)
6	2014	1	1/1	3	19	0/1	Not reported	(17)
7	2014	1	1/1	2	20	0/1	0/1	(18)
8	2019	1	1/1	Not reported	20	0/1	0/1	(19)
9	2020	2	1/2	1.3, 2	1.3, 9	1/1 (with data)	2/2	the present study
total		14	13/14	Median (range) = 2.0 (0.5–5)	Median (range) = 10.0 (1.3–20)	2/7	9/13	

* *In a cohort of 14 patients with primary immunodeficiency syndromes with impaired antibody producton including 4 patients with XLA, six patients were reported to have CSF abnormalities including five pleocytosis. XLA, X-linked agammaglobulinemia; IVIG, intravenous immunoglobulin; CSF, cerebrospinal fluid*.

We found infiltration of CD4^+^ and CD8^+^ T cells in the leptomeninges with a concomitant increase in activated CD4^+^ and CD8^+^ T cells as well as increased levels of inflammatory cytokines, including IFN-γ, IL-6, and IL-8 in the CSF, showing a type I inflammation with the probable involvement of neutrophils and macrophages; however, we did not find any causative microorganism. These observations strongly suggested autoimmunity. Defective B cell tolerance has been described in the human B cells of patients with XLA, which suggested a faulty production of autoantibodies could theoretically occur (27). Mouse B cells lacking the *BTK* gene secrete significantly more IL-12 but much less IL-10 compared to wild type B cells upon Toll-like receptor 9 stimulation (28). The imbalance of these cytokines might cause Th-1 skewed conditions, which could lead to autoimmunity. BTK is present in human monocytes/macrophages. Marron et al. reported that TLR-activated freshly isolated monocytes and monocytoid dendritic cells from patients with XLA produced significantly more TNF-a, IL-6, and IL-10 than control cells from healthy adult individuals, suggesting a role of BTK in the inhibition of TLR-4, TLR-7, and TLR-8-driven cytokine production in primary human cells (29). Indeed, it has been reported that plasma levels of soluble LAG3, indirect indicators of the Th1 activity *in vivo*, were significantly higher in a cohort of 31 XLA cases than healthy controls (30). Hernandez-Trujillo et al. (31) reported that a large portion of XLA cases carried symptoms identical to inflammatory conditions such as arthritis and inflammatory bowel disease. Furthermore, patients with XLA who had developed a variety of autoimmune diseases or chronic inflammatory diseases were reported, which included rheumatoid arthritis, dermatomyositis, autoimmune hemolytic anemia, insulin dependent diabetes, membranoproliferative glomerulonephritis, and Kawasaki disease (32). A structured survey instrument-based study including 783 patients with XLA from 40 centers around the world showed that unusual complications such as inflammatory bowel disease, while individually uncommon, were collectively seen in 20.3% of the patients, suggesting that a broad range of both inflammatory, infectious, and autoimmune conditions could occur in patients with XLA (33). In this study, arthritis and inflammatory bowel disease were reported in 62 patients (7.9%) and 27 patients (3.4%), respectively. A few other inflammatory conditions were listed once, including conjunctivitis, autoimmune enteropathy, cholangitis, autoimmune hemolytic anemia, and sarcoid, although we did not find any of these signs and symptoms in the index patient in our study. When considering the possibility of autoimmunity in our index patient, the normalization of CSF parameters with the improvement of MRI findings and clinical symptoms may have been associated with an immunomodulatory effect of immunoglobulins on the immune dysregulation in the CNS.

Although the genotype-phenotype correlation in XLA accompanying neurodegeneration has not been investigated in previous studies, it should be considered that the *BTK* mutation shared in this family might affect the susceptibility to progressive meningoencephalitis. BTK is involved in signaling of the B cell antigen receptor, mast cell FcεR, IL-5 receptor, and IL-6 receptor, but B cells have only been shown to be vulnerable if functional integrity is disturbed. Although a functional role of BTK has been confined in B lymphocytes so far, a novel role for BTK in neuronal differentiation has been reported (34). Of note, no BTK mutations found in previous patients were identical, except for the patients in the current study. The mutation Val219Leufs^*^9, identified in the current study, was reported in the Chinese registry (35); however, the presence or absence of neurological symptoms were not described.

Regarding the last episode of our index patient that resulted in death, the detection of low copy numbers of VZV DNA in the serum suggested a possible VZV encephalitis; of note, VZV DNA was not detected in the CSF and disappeared from the serum shortly. Importantly, the sensitivity and clinical value of PCR in the context of CSF samples for the detection of intraparenchymal infection, with the exception of HSV encephalitis, remains largely undetermined (36). Therefore, the negative results obtained in this study with respect to the CSF sample, may not be enough to exclude VZV encephalitis as a result of a primary VZV infection. We need to bear in mind that the subject was continuously treated with high dose IVIG and intraventricular immunoglobulin, which might have ameliorated and altered the physical signs, symptoms, and the disease course of a primary VZV infection. Unfortunately, an autopsy was not allowed by the parents; therefore we were unable to make a definitive diagnosis.

In summary, we encountered two patients with XLA who developed progressive meningoencephalitis with no identifiable infectious agent in a family with XLA. The etiology of this rare CNS complications in XLA is currently unknown. Previous studies have suggested a possible association to IVIG, which was clearly refuted in the index case of our study, due to the demonstration of his neurological disorder at presentation. In the future, extensive and unbiased molecular methods to detect causative microorganisms, as well as to investigate the possible role of autoimmunity, are needed to clarify the etiology of CNS complications.

## Data Availability Statement

The original contributions presented in the study are included in the article/Supplementary Material, further inquiries can be directed to the corresponding author/s.

## Ethics Statement

The studies involving human participants were reviewed and approved by Ethical committee, Niigata University School of Medicine. Written informed consent to participate in this study was provided by the participants' legal guardian/next of kin. Written informed consent was obtained from the minor(s)' legal guardian/next of kin for the publication of any potentially identifiable images or data included in this article.

## Author Contributions

CI conceptualized the project. YK and CI wrote the manuscript. HK provided important assays for XLA diagnosis. HS and AK provided histopathological analysis. YK, MI, CS, JU, RH, HI, TT, AS, and CI provided patient care. All authors contributed to the article and approved the submitted version.

## Conflict of Interest

CI reports patent royalty from Juno Therapeutics. The remaining authors declare that the research was conducted in the absence of any commercial or financial relationships that could be construed as a potential conflict of interest.

## References

[1] Rosen FS Cooper MD Wedgwood RJ. The primary immunodeficiencies. N Engl J Med. (1995) 333:431–40. 10.1056/NEJM199508173330707 7616993

[2] Shillitoe B Gennery A. X-linked agammaglobulinaemia: outcomes in the modern era. Clin Immunol. (2017) 183:54–62. 10.1016/j.clim.2017.07.008 28729230

[3] McKinney RE Katz SL Wilfert CM. Chronic enteroviral meningoencephalitis in agammaglobulinemic patients. Clin Infect Dis. (1987) 9:334–56. 10.1093/clinids/9.2.334 3296100

[4] Misbah SA Spickett GP Ryba PCJ Hockaday JM Kroll JS Sherwood C. Chronic enteroviral meningoencephalitis in agammaglobulinemia: case report and literature review. J Clin Immunol. (1992) 12:266–70. 10.1007/BF00918150 1512300

[5] Halliday E. Enteroviral infections in primary immunodeficiency (PID): a survey of morbidity and mortality. J Infect. (2003) 46:1–8. 10.1053/jinf.2002.1066 12504601

[6] Jones TPW Buckland M Breuer J Lowe DM. Viral infection in primary antibody deficiency syndromes. Rev Med Virol. (2019) 29:e2049. 10.1002/rmv.2049 31016825

[7] Ziegner UHM Kobayashi RH Cunningham-Rundles C Español T Fasth A Huttenlocher A. Progressive neurodegeneration in patients with primary immunodeficiency disease on IVIG treatment. Clin Immunol. (2002) 102:19–24. 10.1006/clim.2001.5140 11781063

[8] Staines Boone AT Torres Martínez MG López Herrera G de Leija Portilla JO Espinosa Padilla SE Espinosa Rosales FJ. Gastric adenocarcinoma in the context of X-linked agammaglobulinemia. J Clin Immunol. (2014) 34:134–7. 10.1007/s10875-013-9971-5 24338562

[9] Utsumi J Asami K Sasazaki Y Tanba M Nashimoto A Nemoto K A boy with common variable immunodeficiency complicated with gastric cancer [a paraphrase by the present study; article in Japanese]. Japanese J Pediatr Oncol. (1995) 32:67–70.

[10] Kanegane H Futatani T Wang Y Nomura K Shinozaki K Matsukura H. Clinical and mutational characteristics of X-linked agammaglobulinemia and its carrier identified by flow cytometric assessment combined with genetic analysis. J Allergy Clin Immunol. (2001) 108:1012–20. 10.1067/mai.2001.120133 11742281

[11] Kothur K Wienholt L Brilot F Dale RC. CSF cytokines/chemokines as biomarkers in neuroinflammatory CNS disorders: a systematic review. Cytokine. (2016) 77:227–37. 10.1016/j.cyto.2015.10.001 26463515

[12] Mizutani T Endoh D Okamoto M Shirato K Shimizu H Arita M. Rapid genome sequencing of RNA viruses. Emerg Infect Dis. (2007) 13:322–4. 10.3201/eid1302.061032 17479903 PMC2725858

[13] Shiroma N Omi T Hasegawa H Nagashima K Ohta T. A case of X-linked agammaglobulinemia with progressive encephalitis. Pediatr Neurol. (2004) 31:371–3. 10.1016/j.pediatrneurol.2004.05.007 15519123

[14] Papapetropoulos S Friedman J Blackstone C Kleiner GI Bowen BC Singer C A progressive, fatal dystonia-Parkinsonism syndrome in a patient with primary immunodeficiency receiving chronic IVIG therapy. Mov Disord. (2007) 22:1664–6. 10.1002/mds.21631 17588239

[15] Tuzankina I Kobeleva Y Kiseleva N Bolkov M Reuter G Maródi L. Cytotoxic T lymphocytes mediate neuronal injury in patients with X-linked agammaglobulinemia and progressive neurodegenerative disease. Allergy. (2011) 66:1617–8. 10.1111/j.1398-9995.2011.02718.x 21951217

[16] Mohammadzadeh I Yeganeh M Khaledi M Salehiomran M Aghamohammadi A Rezaei N. Debilitating progressive encephalitis in a patient with BTK deficiency. Acta Microbiol Immunol Hung. (2012) 59:335–42. 10.1556/AMicr.59.2012.3.4 22982637

[17] Domingo A Schmidt TGPM Barcelon E Lukban M Westenberger A Klein C. X-linked agammaglobulinemia with hearing impairment, dystonia-parkinsonism, and progressive neurodegeneration. J Neurol. (2014) 261:2225–7. 10.1007/s00415-014-7483-8 25270678

[18] Sag AT Saka E Ozgur TT Sanal O Ayvaz DC Elibol B. Progressive neurodegenerative syndrome in a patient with x-linked agammaglobulinemia receiving intravenous immunoglobulin therapy. Cogn Behav Neurol. (2014) 27:155–9. 10.1097/WNN.0000000000000037 25237746

[19] Gall T Hawley J Ma Y Progressive neurodegeneration in X-linked agammaglobulinaemia (meeting abstract). Neurology. (2019) 92(15 Supplement):P1.7–005.

[20] Bishu S Madhavan D Perez P Civitello L Liu S Fessler M. CD40 ligand deficiency: neurologic sequelae with radiographic correlation. Pediatr Neurol. (2009) 41:419–27. 10.1016/j.pediatrneurol.2009.07.003 19931163 PMC3130593

[21] Livingston JH Stivaros S Warren D Crow YJ. Intracranial calcification in childhood: a review of aetiologies and recognizable phenotypes. Dev Med Child Neurol. (2014) 56:612–26. 10.1111/dmcn.12359 24372060

[22] Frémond M-L Pérot P Muth E Cros G Dumarest M Mahlaoui N. Next-generation sequencing for diagnosis and tailored therapy: a case report of astrovirus-associated progressive encephalitis. J Pediatric Infect Dis Soc. (2015) 4:e53–7. 10.1093/jpids/piv040 26407445

[23] Quan P-L Wagner TA Briese T Torgerson TR Hornig M Tashmukhamedova A. Astrovirus encephalitis in boy with X-linked agammaglobulinemia. Emerg Infect Dis. (2010) 16:918–25. 10.3201/eid1606.091536 20507741 PMC4102142

[24] Waddell L Pachal N Mascarenhas M Greig J Harding S Young I. Cache Valley virus: A scoping review of the global evidence. Zoonoses Public Health. (2019) 66:739–58. 10.1111/zph.12621 31254324 PMC6851749

[25] Wilson MR Suan D Duggins A Schubert RD Khan LM Sample HA. A novel cause of chronic viral meningoencephalitis: Cache Valley virus. Ann Neurol. (2017) 82:105–14. 10.1002/ana.24982 28628941 PMC5546801

[26] Watanabe S Mizutani T Sakai K Kato K Tohya Y Fukushi S. Ligation-mediated amplification for effective rapid determination of viral RNA sequences (RDV). J Clin Virol. (2008) 43:56–9. 10.1016/j.jcv.2008.05.004 18595773 PMC7108420

[27] Ng Y-S Wardemann H Chelnis J Cunningham-Rundles C Meffre E. Bruton's tyrosine kinase is essential for human B cell tolerance. J Exp Med. (2004) 200:927–34. 10.1084/jem.20040920 15466623 PMC2213290

[28] Lee K-G Xu S Wong E-T Tergaonkar V Lam K-P. Bruton's tyrosine kinase separately regulates NFkappaB p65RelA activation and cytokine interleukin (IL)-10/IL-12 production in TLR9-stimulated B Cells. J Biol Chem. (2008) 283:11189–98. 10.1074/jbc.M708516200 18276597

[29] Marron TU Martinez-Gallo M Yu JE Cunningham-Rundles C. Toll-like receptor 4-, 7-, and 8-activated myeloid cells from patients with X-linked agammaglobulinemia produce enhanced inflammatory cytokines. J Allergy Clin Immunol. (2012) 129:184–90.e1–4. 10.1016/j.jaci.2011.10.009 22088613 PMC3428022

[30] Amedei A Romagnani C Benagiano M Azzurri A Fomia F Torrente F. Preferential Th1 profile of T helper cell responses in X-linked (Bruton′s) agammaglobulinemia. Eur J Immunol. (2001) 31:1927–34. 10.1002/1521-414120010631:6<1927::AID-IMMU1927>3.0.CO 11433390

[31] Hernandez-Trujillo VP Scalchunes C Cunningham-Rundles C Ochs HD Bonilla FA Paris K. Autoimmunity and inflammation in X-linked agammaglobulinemia. J Clin Immunol. (2014) 34:627–32. 10.1007/s10875-014-0056-x 24909997 PMC4157090

[32] Behniafard N Aghamohammadi A Abolhassani H Pourjabbar S Sabouni F Rezaei N. Autoimmunity in X-linked agammaglobulinemia: Kawasaki disease and review of the literature. Expert Rev Clin Immunol. (2012) 8:155–9. 10.1586/eci.11.94 22288453

[33] El-Sayed ZA Abramova I Aldave JC Al-Herz W Bezrodnik L Boukari R. X-linked agammaglobulinemia (XLA): phenotype, diagnosis, and therapeutic challenges around the world. World Allergy Organ J. (2019) 12:100018. 10.1016/j.waojou.2019.100018 30937141 PMC6439403

[34] Yang EJ Yoon J-H Chung KC. Bruton's tyrosine kinase phosphorylates cAMP-responsive element-binding protein at serine 133 during neuronal differentiation in immortalized hippocampal progenitor cells. J Biol Chem. (2004) 279:1827–37. 10.1074/jbc.M308722200 14597636

[35] Lee PPW Chen T-X Jiang L-P Chan K-W Yang W Lee B-W. Clinical characteristics and genotype-phenotype correlation in 62 patients with X-linked agammaglobulinemia. J Clin Immunol. (2010) 30:121–31. 10.1007/s10875-009-9341-5 19904586

[36] Tunkel AR Glaser CA Bloch KC Sejvar JJ Marra CM Roos KL. The management of encephalitis: clinical practice guidelines by the Infectious Diseases Society of America. Clin Infect Dis. (2008) 47:303–27. 10.1086/589747 18582201

