# Supplementary material for: Fatal Progressive Meningoencephalitis Diagnosed in Two Members of a Family With X-Linked Agammaglobulinemia
Source: Front Pediatr. 2020 Sep 18;8:579. doi: 10.3389/fped.2020.00579 (PMC7530192; doi:10.3389/fped.2020.00579)
Supplement: Supplementary file 1 [file Table_1.pdf]

Supplemental table 1: Extensive investigations for possible pathogens conducted in the index patient

pathogen	method	sample	at initial presentation (1y3m)	at the onset of hemiplegia (1y7m)	at the install of Ommaya reservoir (2y3m)	at the onset of generalized convulsion (5y5m)
enterovirus	PCR	CSF	(-)	(-)	(-)	
		serum		(-)	(-)	
		biopsied specimen			(-)	
HHV6	PCR	CSF	(-)	(-)		(-)
		serum	(-)			(-)
HHV7	PCR	CSF	(-)	(-)		(-)
		serum				(-)
HHV8	PCR	serum		(-)		
EBV	PCR	CSF		(-)		(-)
		serum	(-)	(-)		(-)
CMV	PCR	CSF		(-)		(-)
		serum	(-)	(-)		(-)
VZV	PCR	CSF		(-)	(-)	(-)
		serum		(-)	(-)	positive
		biopsied specimen			(-)	
HSV	PCR	CSF	(-)	(-)		(-)
		serum				(-)
JC virus	PCR	CSF	(-)			
parbovirus B19	PCR	CSF	(-)	(-)		
adenovirus	PCR	CSF			(-)	
		serum			(-)	
		biopsied specimen			(-)	
HIV	PCR	plasma		(-)		
Japanese encephalitis virus	PCR	CSF		(-)		
measle virus	PCR	CSF		(-)		
parechovirus	PCR	CSF			(-)	(-)
		serum			(-)	(-)
		biopsied specimen			(-)	
rhinovirus	PCR	CSF	(-)	(-)		
HTLV-1	PCR	serum		(-)		
MAC	PCR	CSF	(-)			
Toxoplasma gondii	PCR	CSF			(-)	
		serum	(-)		(-)	
		biopsied specimen			(-)	
Candida	latex agglutination test	CSF	(-)	(-)		
		serum	(-)	(-)		
Cryptococcus	latex agglutination test	CSF	(-)	(-)		
		serum	(-)	(-)		
Aspergillus	ELISA	CSF	(-)	(-)		
		serum	(-)	(-)		
Mycoplasma pneumoniae	PCR	CSF		(-)		
Bartonella species	PCR	CSF			(-)	
		serum			(-)	
		biopsied specimen			(-)	
culture (bacteria and fungus)		CSF	(-)			
		throat swab	(-)			
		stool	(-)			
		blood		(-)		
virus isolation (culture)		CSF	(-)	(-)		
		throat swab	rhinovirus*1	(-)		
		stool	poliovirus type 2	(-)		

HHV: human herpes virus, EBV: Epstein-Barr virus, CMV: cytomegalovirus, VZV: varicella-zoster virus, HSV: herpes simplex virus, HIV: human immunodeficiency virus, HTLV-1: human T cell leukemia virus type 1, MAC: *Mycobacterium avium complex* , RT-PCR: reverse transcription polymerase chain reaction, ELISA: enzyme-linked immunosorbent assay, CSF: cerebrospinal fluid. *1: The viral cultures were performed at two independent laboratories (commercial laboratory and a local government-funded institute) and reported identical results (rhinovirus only). The method employed are supposed to detect other respiratory viruses including influenza, parainfluenza, enterovirus, RS virus, and so on.
