# Supplementary material for: Fatal Progressive Meningoencephalitis Diagnosed in Two Members of a Family With X-Linked Agammaglobulinemia
Source: Front Pediatr. 2020 Sep 18;8:579. doi: 10.3389/fped.2020.00579 (PMC7530192; doi:10.3389/fped.2020.00579)
Supplement: Supplementary file 2 [file Presentation_1.pdf]

Supplementary Material

1 Supplementary Figures

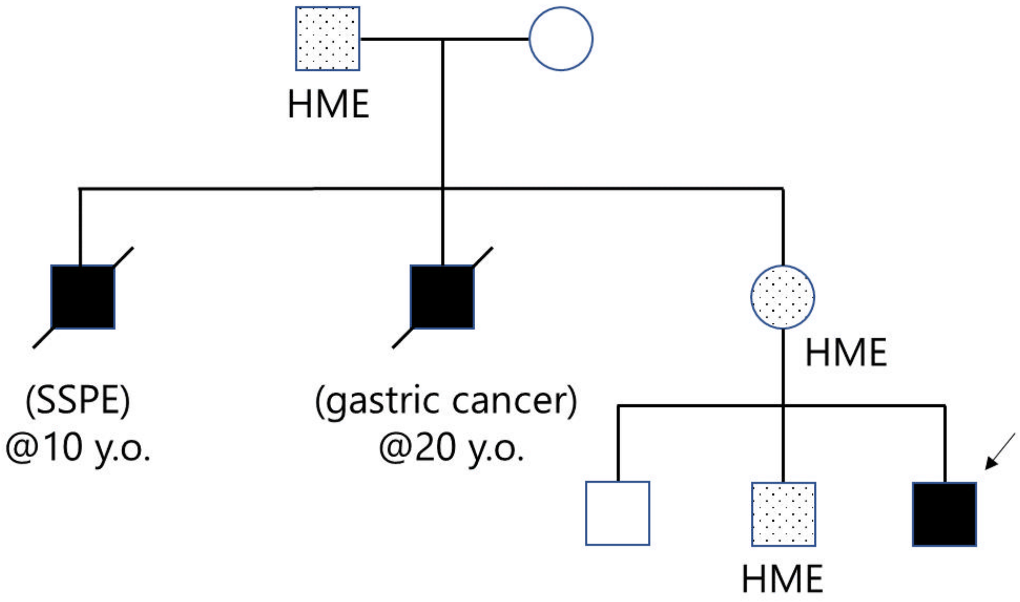

Supplementary Figure 1. A black square denotes individuals with XLA. A diagonal line indicates that a person is dead of a disease denoted in the parenthesis. A dotted square or circle denotes a person affected by hereditary multiple exostosis (HME). A square and a circle indicate male and female member, respectively. The arrow depicts the index patient.
